# Prediction of non-muscle-invasive bladder cancer recurrence by measurement of checkpoint HLAG’s receptor ILT2 on peripheral CD8^+^ T cells

**DOI:** 10.18632/oncotarget.26036

**Published:** 2018-09-04

**Authors:** Francois Desgrandchamps, Joel LeMaoult, Annabelle Goujon, Adrien Riviere, Antonio Rivero-Juarez, Malika Djouadou, Amory de Gouvello, Clement Dumont, Ching-Lien Wu, Stephane Culine, Jerome Verine, Nathalie Rouas-Freiss, Christophe Hennequin, Alexandra Masson-Lecomte, Edgardo D. Carosella

**Affiliations:** ^1^ CEA, DRF-Francois Jacob Institute, Research Division in Hematology and Immunology (SRHI), Saint-Louis Hospital, Paris, France; ^2^ AP-HP, Saint-Louis Hospital, Department of Urology, Paris, France; ^3^ University Paris Diderot, Sorbonne Paris Cité, UMR E_5 Institut Universitaire d’Hématologie, Saint-Louis Hospital, Paris, France; ^4^ Infectious Diseases Unit, Instituto Maimonides de Investigación Biomédica de Córdoba (IMIBIC), Hospital Universitario Reina Sofía, Universidad de Córdoba, Córdoba, Spain; ^5^ AP-HP, Saint-Louis Hospital, Department of Medical Oncology, Paris, France; ^6^ AP-HP, Saint-Louis Hospital, Department of Pathology, Paris, France; ^7^ AP-HP, Saint-Louis Hospital, Department of Radiotherapy, Paris, France

**Keywords:** ILT2, HLA-G, immune checkpoint, bladder, cancer

## Abstract

**Background and Objective:**

Recurrence of non-muscle invasive bladder cancer (NMIBC) after initial management occurs in 60–70% of patients. Predictive criteria for recurrence remain only clinical and pathological. The aim of this study was to investigate the prognostic significance of the proportion of checkpoint HLA-G’s receptor ILT2-expressing peripheral CD8^+^ T cells.

**Results:**

The proportion of CD4^+^ILT2^+^and CD8^+^ILT2^+^ T cells was not increased in NMIBC compared to controls. However, a strong association was found between recurrence and CD8^+^ILT2^+^ T cell population levels (*p* = 0.0006). Two-year recurrence-free survival was 83% in patients with less than 18% CD8^+^ILT2^+^ T cells, 39% in the intermediary group, and 12% in patients with more than 46% CD8^+^ILT2^+^ T cells. Multivariate analyses demonstrated that the proportion of CD8^+^ILT2^+^ T cells was an independent predictive factor for recurrence. Adding CD8^+^ILT2^+^ T cells population level to clinical variables increased the predictive accuracy of the model by 4.5%.

**Materials and Methods:**

All patients treated for NMIBC between 2012 and 2014 were included prospectively. Blood samples, tumor and clinico-pathological characteristics were collected. HLA-G expression was measured using IHC, and CD8^+^ILT2^+^ T cell levels using flow cytometry. Association between HLA-G and CD8^+^ILT2^+^ T cell population levels with NMIBC risk of recurrence was investigated using Cox regression analyses. Prediction was measured using the concordance index statistic.

**Conclusions:**

We demonstrated a strong association between the proportion of circulating CD8^+^ILT2^+^ T cells and NMIBC risk of recurrence. Gain in prediction was substantial. If externally validated, such immunological marker could be integrated to predict NMIBC recurrence.

## INTRODUCTION

Bladder cancer is the second most common genitourinary tract malignancy worldwide [1]. 75% to 85% of patients are initially diagnosed with non-muscle invasive disease (NMIBC) [2]. NMIBC is managed by transurethral resection of the bladder with or without intravesical therapy (Mytomicine C or BCG), depending on clinicopathological assessment of the risk of recurrence and progression. Recurrence occurs in 60–70% of patients and 10–15% progress to muscle-invasive disease within 5 years [3, 4].

To predict the short- and long-term probabilities of disease recurrence and progression, two different scoring systems and risk tables were developed [4–6] based on clinical and pathological parameters by the European Organization for Research and Treatment of Cancer (EORTC) and by the Spanish Urological Club for Oncological Treatment (CUETO). Unfortunately, if these score accurately predict progression, they are known to poorly predict and overestimate recurrence, leading to stringent follow-up and alteration of the patients’ quality of life [7–12].

The inhibition of immune checkpoints (in particular PD1/PD-L1 and CTLA4) is a promising therapeutic approach for the management of cancer. It has already been approved in some cancers, and is being evaluated in the context of genitourinary cancers including bladder [13]. HLA-G is an immune checkpoint molecule well known for its tolerogenic role in maternal-fetal tolerance, and that is commonly neo-expressed by solid tumors (including bladder cancer [14, 15]). HLA-G expression by solid tumors has been associated with worse prognosis and higher grade in numerous cancers including but not restricted to kidney, ovary, breast, lung, colon [16]. Its main receptors are LILRB1 and LILRB2 (also known as ILT2/CD85j and ILT4/CD85d) and KIR2DL4. These are differentially expressed by immune cells, ILT2 being the receptor that is expressed on some T lymphocytes [17]. Through its receptors, HLA-G inhibits the cytolytic function of NK cells, cytotoxic T lymphocytes (CTLs) and γ/δT cells, the alloproliferative response of CD4^+^ T cells, the proliferation of T cells and peripheral blood NK cells, the maturation and function of dendritic cells (DCs). HLA-G is also known to induce regulatory T cells and myeloid suppressive cells. Thus, HLA-G is capable of inhibiting all actors of anti-tumor responses and in contrast to both CTLA-4 and PD-1, of blocking all stages of such an anti-tumor response, from the APC activation and effector priming, to the function of a fully activated cytotoxic T cell or NK cell. However, because ILT2 is normally only expressed by a minority of CD4^+^ T cells (0–4%) and CD8^+^ T cells (5–20%), variations in its expression might impact HLA-G capability to inhibit anti-tumor effectors. In the current study, we investigated whether the proportion of immune effectors expressing ILT2 might be associated with recurrence in NMIBC, in relation or not with HLA-G expression at the tumor site.

## RESULTS

Seventy-six patients matched the inclusion criteria. The characteristics of patients and aged-matched controls are summarized in Table 1. Mean and median follow-up of the patient cohort were 20.05 ± 7.55 and 20.02 [13.87–26.87] months respectively. During follow up, recurrence occurred in 31 (40.8%) patients.

**Table 1:** Patient and tumor characteristics

	NMIBC patients *N* = 76	Age-matched controls *N* = 20
Age median [95% IQR] mean (SD)	70.0 [56.7–84.0] 70.3 (9.7)	68.5 [47–78] 68.5 (8.9)
Gender Male Female	61 (80.3%) 15 (19.7%)	17 3
Tobacco Use Smoker Past smoker Never Smoker Incident/Prevalent Previous adjuvant therapy (BCG or Mitomycin C) Previous BCG instillation Stage and Grade Ta G1 Ta G2 Ta G3 T1 G2 T1 G3 Tis	20 (26.3%) 40 (52.6%) 16 (21.1%) 42 (55.3%)/34 (44.7%) 27 (35%) 13 (17.1%) 8 (10.5%) 29 (38.2%) 8 (10.5%) 10 (13.1%) 16 (21.1%) 5 (6.6%)	
Multiplicity Unique Multiple Plane lesion	40 (52.6%) 33 (43.4%) 3 (3.9%)	
EORTC score ≤4 5–9 ≥10 CD8–ILT2 median [75% IQR] mean (SD)	30 (39.5%) 34 (44.7%) 12 (15.8%) 32.4 [17.4–45.9] 32.7 (18.1)	38 [2.7–75.5] 36.8 (22.6)
Number of patients with tumor recurrence Number of patients with progression	31 (40.8%) 4 (5.3%)	

**Figure 1:**
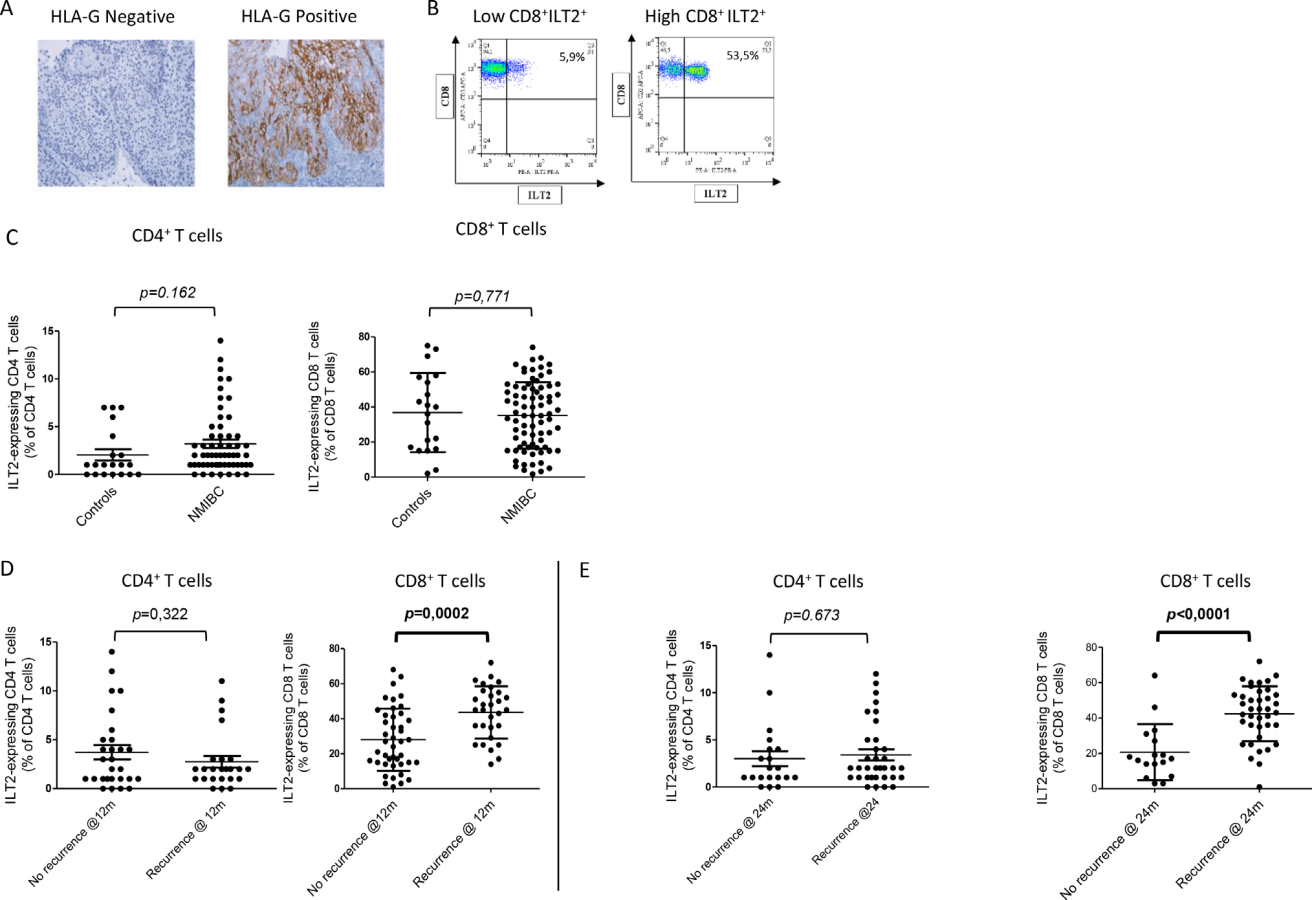
(**A**) HLA-G expression in NMIBC biopsies. Two representative results obtained are shown. Brown labelling indicates HLA-G positivity. (**B**) Representative images of the results obtained for ILT2 cell-surface expression on CD8^+^ T cells from NMIBC patients with low (left) and high (right). Proportion of the ILT2-positive population within the CD8^+^ T cell population is indicated. (**C**) ILT2 expression levels on peripheral CD3^+^CD4^+^ T cells and CD3^+^CD8^+^ T cells for 25 healthy donors (HD), 20 aged-matched controls (Aged-matched), and 27 (for CD4^+^ T cells) or 76 (for CD8^+^ T cells) NMIBC patients. (**D**) Proportion of ILT2^+^ peripheral CD3^+^CD4^+^ T cells and CD3^+^CD8^+^ T cells at the time of inclusion from NMIBC patients who recurred and did not recur within 12 months. (**E**) Proportion of ILT2^+^ peripheral CD3^+^CD8^+^ T cells at the time of inclusion from NMIBC patients who recurred and did not recur within 24 months. Mean and standard deviation are shown. *P* was calculated using Mann–Whitney test.

Immunohistochemistry analyzes revealed HLA-G expression in 31.5% of tumor biopsies (Figure 1A), but no association between HLA-G expression and recurrence was found (*p* = 0.721, data not shown). Similarly, no association was found between soluble plasma HLA-G levels and recurrence (*p* = 0.950, data not shown).

We compared the percentage of CD4+ and CD8+ T cells expressing HLA-G receptor ILT2 in NMIBC and in age-matched controls. We observed a high variability in the proportions of ILT2-expressing T cells, especially in the CD8^+^ T cell compartment. A representative example of the results obtained for low and high CD8^+^ILT2^+^ T cell proportions is shown on Figure 1B. However, no significant difference was observed between these two populations (*p* = 0.162 for CD4^+^ T cells, and *p* = 0.771 for CD8^+^ T cells), indicating that ILT2 expression by CD4^+^ and CD8^+^ T cells is not associated with NMIBC risk (Figure 1C).

We then analyzed whether the proportion of ILT2-expressing T cells was associated with recurrence. Recurrence was not associated with CD4^+^ILT2^+^ T cell proportions at any time point (*p* = 0.322). However recurring patients showed significantly higher proportions of CD8^+^ILT2^+^ T cells compared to non-recurring patients at both 12 and 24 months (43.6 ± 2.7% vs 28.3 ± 2.7%, *p* = 0.0002, Figure 1D, and 42.4 ± 2.5% vs 21.7% ± 3.7%, *p* < 0.0001, Figure 1E).

**Figure 2:**
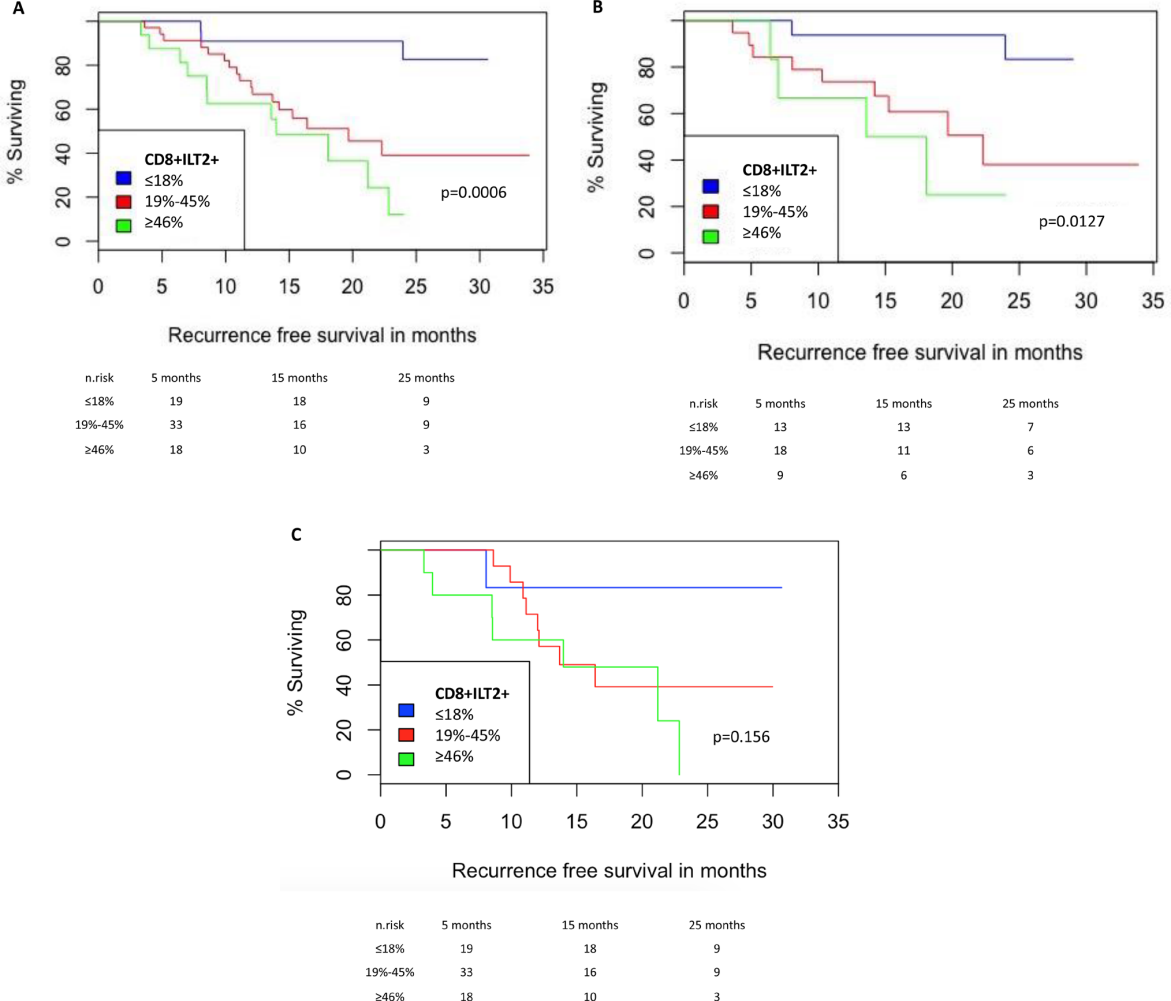
NMIBC Recurrence-free survival according to the proportion of CD8^+^ILT2^+^ T cells (**A**) Analysis for the whole cohort. (**B**) Analysis for incident patients. (**C**) Analysis for prevalent patients. Recurrence-free survival curves are shown for Low (≤18% CD8^+^ILT2^+^ T cells among CD8^+^ T cells) vs intermediate (19%–45% CD8^+^ILT2^+^ T cells among CD8^+^ T cells) vs high (≥46% CD8^+^ILT2^+^ T cells among CD8^+^ T cells).

Using Kaplan–Meier analysis, 2-years recurrence free survival according to CD8^+^ILT2^+^ T cell proportions were 82%, 39% and 12% respectively (Figure 2A, *p* = 0.0006). Cox regression was applied to assess factors associated with recurrence (Table 2). In univariate analysis, high EORTC score, multiplicity, concurrent CIS and CD8^+^ILT2^+^ T cell population proportions were associated with recurrence. In multivariate analysis, Concurrent CIS (HR 4.83, *p* = 0.006), multiplicity (HR 2.46, *p* = 0.02) and CD8^+^ILT2^+^ T cell population proportions (HR 7.86 for high level patients, *p* = 0.02) remained significant.

**Table 2:** Cox regression for factors associated with tumor recurrence

	Unadjusted			Adjusted					
	HR	CI	*p*-value	HR		CI	*p*-value		
Age	0.99	[0.96–1.03]	0.88						
Gender	0.76	[0.33–1.78]	0.53						
EORTC score ≤4 5–9 ≥10	Ref 0.87 2.48	- [0.39–1.94] [0.97–6.36]	- 0.74 0.05						
Concurrent CIS No Yes	*Ref* **3.38**	**-** **[1.29–8.85]**	**-** **0.01**	*Ref* 4.82	- **[1.55–14.99]**			- 0.006	
Grade G1 G2 G3	*Ref* 1.25 0.90	- [0.38–4.29] [0.25–3.27]	- 0.71 0.88						
pT Stage pTa pT1 Isolated CIS	*Ref* 0.95 2.92	- [0.41–2.17] [0.98–8.67]	- 0.90 0.05						
Incident/Prevalent	1.73	[0.88–3.54]	0.12						
Multiplicity	**2.76**	**[1.32–5.76]**	**0.007**	2.46	**[1.12–5.39]**				0.02
Ratio CD8 – ILT2 ≤20% 21%–47% ≥48%	*Ref* **5.48** **9.12**	- **[1.59–18.88]** **[2.50–33.19]**	- **0.007** **0.0008**	*Ref* **8.24** **7.86**	**-** **[2.23–30.44]** **[2.12–29.04]**				**-** **0.01** **0.02**

Because prevalent patients had undergone surgical procedures, tumor removal(s), and possibly BCG immunotherapeutic instillations, we next investigated whether CD8^+^ILT2^+^ T cell population proportions were different, and whether the association of CD8^+^ILT2^+^ T cell population proportions with recurrence applied to incident and/or prevalent patients. The same analysis as above was therefore performed on incident and prevalent cohorts, counting 42 and 34 individuals, respectively (Figure 2B and 2C). No differences in CD8^+^ILT2^+^ T cell population proportions were observed between incident and prevalent cohorts. Interestingly, the association between CD8^+^ILT2^+^ T cell proportions and recurrence held true for the incident cohort (*p* = 0.0127), but not for the prevalent cohort (*p* = 0.156) despite the same trend being clearly observable.

Concordance index calculation at different time points was performed to estimate the predictive ability of a model with and without CD8^+^ILT2^+^ T cell proportion levels (Table 3). At 30 months of follow up, the clinical variables alone predicted 69.6% of the risk of recurrence while CD8^+^ILT2^+^ T cell proportions predicted 63.4%. Adding CD8^+^ILT2^+^ T cell proportions to the clinical variables increased the c-index to 74.1% (4.5% upgrade).

**Table 3:** Calculation of the concordance index at different time points of the model with clinical variables alone, with CD8-ILT2 alone and with both clinical variables and CD8-ILT2

	T = 5 months	T = 10 months	T = 15 months	T = 20 months	T = 25 months	T = 30 months
Clinical variables^*^	69.1	79.7	71.2	70.9	69.6	69.6
CD8-ILT2	70.3	62.7	62.5	63.5	64.3	64.3
Clinical variables + CD8-ILT2	78.6	80.7	74.9	75.2	74.1	74.1

^*^EORTC categories, Multiplicity, Concurrent CIS.

## DISCUSSION

Evading immune response is one of the hallmarks of cancer [18], assessing both the extent and the type of tumor-related inflammatory response could have major prognosis implications. While interest has been focused in the past years on the PD1/PDL1 checkpoint due to positive results of clinical trials [19], other checkpoints inhibition might provide as good a clinical benefit. As stated earlier, HLA-G expression by solid tumors has been associated with worse prognosis and higher grade in numerous cancers including kidney, ovary, breast, lung, colon [16]. However, no association was found in bladder cancer in two small studies [14, 15], but this could be due to the fact that only HLA-G expression was investigated, and not that of ILT2, without which HLA-G cannot inhibit T cells [20]. Thus, in this study, we investigated whether HLA-G expression by the tumor and/or in plasma, and ILT2 expression by the peripheral T cells, taken independently or together, was of interest for the prognosis prediction of NMIBC. Although HLA-G was strongly expressed in one third of tumors, we confirmed published data [14, 15] failing to demonstrate its association with NMIBC prognosis. Similarly, plasma levels of soluble HLA-G were not associated with recurrence. In this respect NMIBC differs from most solid tumors [16]. We found that the proportion of CD4^+^ILT2^+^ T cells among CD4^+^ T cells was not associated with recurrence, but that the proportion of peripheral CD8^+^ILT2^+^ T cells among CD8^+^ T cells was an independent prognosis marker for NMIBC.

Association between various types of inflammatory markers and bladder cancer prognosis has been extensively published in the literature [21]. The prognostic significance of serum levels of proinflammatory mediators such as IL-6, CRP and TNFalpha has been demonstrated in multiple cancers [22, 23]. CD8^+^ T cell count was assessed as a blood marker using flow cytometry in one study [24]. Authors observed that peripheral blood CD8^+^ T cell count was inversely correlated to tumour infiltration with CD8^+^ T cells (*r*^2^ = 0.63, *p* < 0.0001). Low levels of CD8^+^ T cells in blood were associated with lower intravesical recurrence after TURB applying a multivariable analysis (HR = 0.4, 95% CI 0.17–0.94).

Whereas optimizing prognostic assessment is required for personalized treatment, most of the prognostic studies in bladder cancer have been limited by methodological bias. Because demonstrating a statistical association does not provide information about the gain in prediction, the use of promising molecular prognostic markers has yet not been applied in daily practice [25]. Using the concordance index statistic, we demonstrated that adding ILT2 level to clinical prognosticators increased prediction of recurrence risk by 4.5%. Measuring CD8^+^ILT2^+^ T cell levels in the peripheral blood can easily be performed in routine, as it can be automated and only requires a blood sample.

In our study, follow-up of patients after initial CD8^+^ILT2^+^ T cell population proportion determination was short. This was intentional. Indeed, our hypothesis was that the proportion of CD8^+^ILT2^+^ T cells could be a predictor of disease recurrence. However, it was also our hypothesis that, by definition, this proportion was not stable over time. Thus, drawing an association between the level of an immune marker and an outcome observed years afterwards is, to our point of view, hazardous and actually constitutes one of the main problems encountered with prediction of response to immunotherapy using immunohistochemistry on the initial tumor at the time of metastases. In the present context for instance, worsening of the disease, or efficacy of a treatment, that obviously affect patient status and prognosis, should modify a parameter correlated with recurrence such as CD8^+^ILT2^+^ T cell population proportion. It is therefore important to perform longitudinal studies in order to determine how stable CD8^+^ILT2^+^ T cell population proportions are, if they vary with changes in disease status, and if they can reliably be used in patient monitoring, to predict disease changes, or treatment efficacy for instance.

The biological function of peripheral CD8^+^ILT2^+^ T cells still needs to be clarified. It is very likely that they are a diverse subset, containing CD8^+^ T cells of various specificities and possibly functions. Expanded CD8^+^ILT2^+^ T cells could be specific for the bladder tumor exerting anti-tumor control but could also be regulatory cells, leading to poor tumor immune control and recurrence. Such CD8^+^ suppressor cells have been described in other contexts [26]. Moreover, CD8^+^ILT2^+^ T cells may not be tumor-specific, but specific for antigens unrelated to the bladder tumor. In this case, their expansion to up to 80% of the peripheral CD8^+^ T cells would cause an overall reduction of the CD8^+^ T cell antigenic repertoire (including potential anti-tumor specificities) that could also be linked to poorer CD8^+^ T cell responses [27]. These three situations may happen concomitantly, and this might explain why a simple association between HLA-G expression, ILT2 expression and recurrence is not observed. In general, the mechanisms responsible for checkpoint inhibition and anti-tumor response are still poorly understood, and biomarkers of response to immunotherapy are still lacking. It is therefore important to better characterize this CD8^+^ILT2^+^ T cell population in order to understand why it is associated with recurrence in NMIBC.

Our study has limitations. The size of our cohort is small and follow-up is limited. However, most patients recurred in a short interval and the high number of recurrence allows a sufficient number of events to perform multivariate analysis with proper power. While subgroup analyses are not powerful enough to drive clear conclusions, the absence of association in the prevalent cohort compared to the incident one suggest that previous treatment such as BCG therapy probably influence CD8^+^ T cell levels, biasing the analyses. Finally, the absence of external validation is likely leading to an overestimation of the results. This is the main limitation of most biomarker studies published in the literature. External validation is time and resource consuming but should be the ultimate goal of any biomarker study.

## MATERIALS AND METHODS

### Ethical considerations

All patients and age-matched controls gave written informed consent, and the study was approved by the local institutional board.

### Population

We designed a prospective longitudinal study including all consecutive patients treated for a NMIBC at our institution between 2012 and 2014. Both incident (first tumor) and prevalent (history of previous NMIBC) tumors were included. Inclusion criteria were: i) no immunomodulator medication; ii) free of infectious diseases; iii) free of other active or recent (<5 years) tumors other than bladder cancer; iv) no previous or current radiotherapy or chemotherapy; v) no autoimmune disease. Age-matched patients admitted to the Urology department for planned, non-carcinological surgery (resection of prostatic adenoma or treatment of pelvic floor dysfunction) were included as control. Patients with a personal history of cancer, chronic viral infection (other than CMV or EBV) or autoimmune disorder, as well as patients with evidence of cancer on surgical specimens, were excluded.

After first TURBT, patients were classified as low, intermediate or high risk as recommended by the EAU and discussed in the tumor board of the institution. Re-TUR was recommended for all T1 tumors and for Ta high grade depending on the size and the presence of muscle. Intermediate-risk tumors were advised to receive 8 Mitomycin C instillations while high-risk tumors were treated with BCG induction plus 1 year of maintenance. At the end, among the 39 high risk patients included (all G3, all T1), 17 second-looks (43%) were performed and 20 patients received BCG + maintenance (51%).

### Sample/data collection

After retrieval of informed consent, blood samples were collected the day before surgery on EDTA tubes. After TURBT, the pathologist selected one FFPE block representative of each tumor.

The following data were collected prospectively: i) clinical characteristics: age, gender, ethnicity, smoking status, previous history of bladder cancer (prevalent tumors), previous history of intra-vesical instillations; ii) tumor characteristics: T stage, grade according to the 1973 ad 2004 WHO classification, size, concurrent CIS, multiplicity.

EORTC clinical score was calculated according to the EAU guidelines [4].

### Immunohistochemistry and flow cytometry

Tumor HLA-G expression was evaluated by immunohistochemistry on resection biopsies as described elsewhere [28]. Briefly, after selection of one FFPE block representative of each tumor, 4-µm-thick sections were performed and stained using a murine anti-4H84 antibody (an IgG1 recognizing alpha1 domain common to all HLA-G isoforms, dilution 1/200, Santa Cruz Biotechnology, Santa Cruz, CA). The staining was performed on automated slide stainers from Roche (BenchMark ULTRA system, Tucson, AZ) using the OptiView DAB IHC Detection Kit (Roche), Cell Conditioning 1 (CC1) standard antigen retrieval, an antibody incubation time of 32 min at 37° C, ultraWash procedure, counterstaining with Hematoxylin II for 4 min and bluing reagent for 8 min. The immunohistochemical analyses were performed by an uropathologist using a BX51 microscope (Olympus France S.A.S, Rungis). Each immunostaining was scored on the basis of membranous and/or cytoplasmic staining by both staining intensity as low, moderate or strong and the location of staining in papillary urothelial neoplasms and/or contingents of invasive urothelial carcinoma. HLA-G immunostaining was defined as positive if >1% of tumor cells expressed HLA-G. A trophoblastic tissue was used as the positive control and isotype-specific immunoglobulins were used for negative controls with each run.

In all cases (except for healthy controls) blood sampling was performed upon admission to the Urology department prior to surgery. After sampling, plasma was stored at –80° C and peripheral blood mononuclear cells (PBMCs) were isolated using Ficoll (LifeSciences) as per the manufacturer’s instructions and stored at –150° C. Plasma HLA-G was measured by Luminex exactly as previously described [29].

For ILT2 cell-surface expression, FcR were first blocked with human polyclonal immunoglobulin, and cells were stained with fluorochrome-conjugated primary antibodies. Acquisition was made on a MACSQuant 10 flow cytometer (Miltenyi biotec); analysis was performed using the MACSQuantify software (Miltenyi biotec) and Flowjo software (TreeStar).

The following antibodies were used for cell surface staining and phenotyping: from Miltenyi Biotec: CD3-PerCP, CD4-VioBright-FITC, CD8-APC-Vio770, CD19-APC eBioscience: ILT2-PE (Clone HP-F1). Appropriate isotypic controls were systematically used, and ILT2 expression levels on CD19^+^ B cells were used as ILT2 positive staining quality control. CD4^+^ILT2^+^ and CD8^+^ILT2^+^ T cell population levels were expressed as a percentage of the CD3^+^CD4^+^ and CD3^+^CD8^+^ T cells, respectively.

### Follow up

Patients were followed according to the EAU guidelines [30], with regular flexible cystoscopy, urines cytology and CT scan. Recurrence was defined as the occurrence of a new NMIBC, confirmed by histology. Progression was defined as the occurrence of a muscle invasive tumor, appearance of metastasis, or death from bladder cancer.

### Statistical analyses

Continuous variables are expressed as medians and quartiles (Q1–Q3), and were analyzed using the Mann–Whitney *U* test. Categorical variables are expressed as numbers of cases and percentages. CD8^+^ILT2^+^ level was categorized according to 25% and 75% IQR values. The time-to-event was computed as the number of months from TURBT date to tumor recurrence. Censored time was defined as time-to-event. Survival curves were plotted using the Kaplan–Meier method, and compared using the log-rank test. Cox regression analysis adjusted for classical prognosticators was computed to assess association between CD8^+^ILT2^+^ T cell proportions and recurrence. Predictive accuracy of clinical variables and immunological parameters was assessed using the concordance index (pec package). Statistical analyses were performed using the R software v3.2.2. All statistical tests were two-sided, and a *p*-value less than 0.05 was considered significant.

## CONCLUSIONS

At a time where immunotherapy is completely re-designing cancer treatment, we found a strong association between the proportions of CD8^+^ T cells expressing the checkpoint receptor ILT2 and NMIBC risk of recurrence. Adding CD8^+^ILT2^+^ T cell proportion measurement to clinical prognosticators allowed a significant gain in prediction. If externally validated, such immunological markers could be integrated to NMIBC recurrence prediction.
